# A Dangerous Practice

**Published:** 1875-05

**Authors:** 


A Dangerous Practice. — Actual contact with the person affected, or with matter from his mouth or throat, is a sure means of communicating diphtheria. With this in view, the Board of Health urge that children should not be permitted to visit those who are sick with diphtheria and kindred throat diseases. They strongly reprobate the absurd and common practice of urging little children to kiss every visitor. They intimate that diseases other than diphtheria are communicated in this way. Kissing is the common salutation among ladies. It is not confined to dearest friends, and is not accepted as a token of special regard. In fact they don't mean anything by it; but it sometimes amounts to more than they anticipate.

As an instance of what has happened and what may occur again, we recall the following: Many of the young ladies in a female seminary in this city were troubled with sore lips. The affection became so general that a physician was called. He found nothing less than an ulcer, of a specific nature, on the lip of each of the unfortunate girls. And it all came about in a very easy and natural way. The teacher was in the habit of kissing her pupils. The teacher had a beau and it so happened that she sometimes kissed him. He had a sore on his lip and in due time she had a sore on her lip; and, as we have already seen, she passed it around among her pupils.

An example like this ought to be sufficient to show to ladies the folly and danger of promiscuous kissing. Mothers should be careful how they allow their children to kiss other children, or visitors, or casual callers.

It is not improbable that diseases are communicated in this way in many cases where their origin is unknown.

